# Community awareness of childhood arthritis in the UK

**DOI:** 10.1093/rap/rkad099

**Published:** 2024-01-22

**Authors:** Richard P Beesley, Rebecca M Beesley

**Affiliations:** Juvenile Arthritis Research, Tonbridge, UK; Centre for Epidemiology Versus Arthritis, The University of Manchester, Manchester Academic Health Science Centre, Manchester, UK; Juvenile Arthritis Research, Tonbridge, UK

**Keywords:** awareness, juvenile idiopathic arthritis, ethnicity, inequality

## Abstract

**Objective:**

The aim was to measure the level of community awareness in the UK that children and young people can develop arthritis.

**Methods:**

An online survey of a representative quota sample of 2044 adults aged 16–75 years in the UK was conducted between 10 and 13 February 2023 by Ipsos UK, a leading social and market research organization, with participants giving informed consent before taking part. Participants were asked which age band they thought is the earliest that someone can get arthritis. They were also asked whether a series of statements about arthritis were true or false, in addition to a series of demographic questions. Data were weighted to the known population proportions for adults aged 16–75 years in the UK.

**Results:**

Overall, 40% of respondents indicated they believed the earliest that someone could get arthritis was <16 years of age. This was higher amongst respondents with arthritis themselves or if they knew someone with arthritis. Only 19% of respondents were aware that children <5 years of age can get arthritis. This varied by gender and ethnicity (males and minority ethnic groups were less likely to be aware). Minority ethnic groups were also more likely to believe a series of incorrect assumptions to be true.

**Conclusion:**

Awareness that children and young people can get arthritis is low, and it is lower amongst minority ethnic groups. Further research to understand this is important, to enable targeted interventions and awareness-raising resources to be developed and applied as appropriate.

## Graphical Abstract

**Graphical Abstract:**
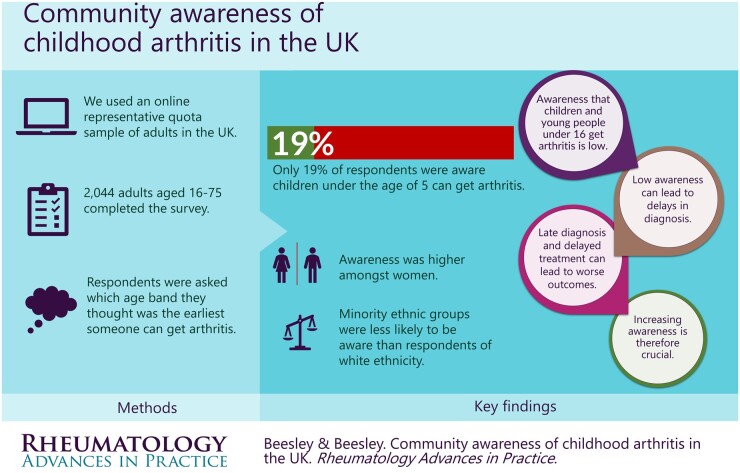

Key messages

- Awareness that children and young people may get arthritis is low.
- Ethnic minority groups are less likely to be aware of childhood arthritis.
- Misconceptions about arthritis are common, particularly amongst minority ethnic groups.

## Introduction

JIA is a heterogeneous group of autoimmune disorders characterized by chronic joint inflammation of unknown aetiology with onset at <16 years of age [1]. This childhood-onset arthritis can be associated with joint erosion, chronic pain and lifelong disability and with extra-articular manifestations, including uveitis. This has implications for physical and mental health [2].

Estimates of incidence and prevalence of JIA vary between countries and study methods [3]. Recent analysis of electronic primary care records in the UK provided updated estimates of incidence of 5.6 per 100 000 population and prevalence of 43.5 per 100 000 population (∼1 in 1600 children <16 years of age) [4].

Early diagnosis is associated with better outcomes [5], and there is a window of opportunity in the early treatment of JIA [6] that is associated with improved long-term outcomes. However, prompt presentation to primary care and prompt specialist referral rely on awareness of JIA in both the community and primary care [7], the latter being a further issue outside the scope of the present study.

Awareness that children and young people can get arthritis is therefore important in early identification, diagnosis and treatment. However, levels of community awareness have not previously been reported in the UK context.

## Methods

Ipsos UK, on behalf of UK charity Juvenile Arthritis Research, conducted an online survey about awareness of childhood arthritis among a quota sample of 2044 adults aged 16–75 years from their national UK-wide Panel, representative for the UK population in terms of age, geographical region and ethnicity. Fieldwork was between 10 and 13 February 2023.

Participants were asked in which age band (<5, 5–10, 11–15, 16–30 years, then 10-year age bands up to 81+ years) is the earliest that someone can get arthritis. They were also asked whether a series of statements about arthritis were true or false, in addition to a series of demographic questions (Supplementary Data S1, available at *Rheumatology Advances in Practice* online).

Data were weighted to the known population proportions for adults aged 16–75 years in the UK.

This work was carried out in accordance with the requirements of the international quality standard for market research, the MRS Code of conduct, ISO 20252, and participants gave informed consent prior to taking part.

## Results

A total of 2044 adults aged 16–75 years in the UK completed the survey online (Table 1). A total of 18% of respondents reported having arthritis themselves, and 55% claimed to know an adult with arthritis. The proportion of respondents who reported having arthritis increased with age, broadly in line with national estimates of prevalence of musculoskeletal disorders [8]. In contrast, only 3% said they knew a child ≤15 years with arthritis, although this was higher amongst parents of children aged ≤17 years living in their household (7%).

**Table 1.** Characteristics of survey participants

Characteristics		
*n*		2044
Gender, *n*	Male	967
	Female	1068
Age band, *n*	16–24 years	272
	25–34 years	338
	35–44 years	370
	45–54 years	406
	55–75 years	658
Children <17 years of age in household		
	Yes	618
	No	1426
Ethnicity		
	White	1727
	Ethnic minority group	287
Experience of arthritis		
	Have arthritis	351
	Know someone with arthritis	1181
	Do not know anyone with arthritis	583
Region of UK, *n*		
	North	489
	Midlands	525
	South	437
	Greater London	265
	Wales	100
	Scotland	176
	Northern Ireland	52

Data presented are unweighted.

### Age of onset of arthritis

Overall, 40% of respondents indicated that they believed the earliest age range that someone could get arthritis was ≤16 years (Table 2). This was higher amongst female respondents (47% *vs* 33% of males, *P* < 0.001), older respondents (52% of those aged 45–75 years *vs* 29% of those aged 16–44 years, *P* < 0.001) and those from a White ethnic group (43%, compared with 23% among ethnic minority groups, *P* < 0.001).

**Table 2.** Awareness that children get arthritis, and knowledge of key statements about arthritis, by gender and ethnicity

Question	Overall	Gender			Ethnicity		
		Male	Female	***P-value*** ^a^	White	Minority ethnic group	***P-value*** ^b^
*n*	2044	1012	1023		1744	273	
Which of the following age ranges do you think is the EARLIEST someone can get arthritis?, *n* (%)							
<5 years old	393 (19)	140 (14)	251 (25)	*P* < 0.001	372 (21)	14 (5)	*P* < 0.001
5–10 years old	251 (12)	106 (10)	143 (14)	*P* = 0.016	226 (13)	25 (9)	n.s.
11–15 years old	181 (9)	90 (9)	90 (9)	n.s.	156 (9)	23 (8)	n.s.
16–30 years old	436 (21)	225 (22)	211 (21)	n.s.	380 (22)	50 (18)	n.s.
31–40 years old	269 (13)	136 (13)	132 (13)	n.s.	202 (12)	67 (24)	*P* < 0.001
41–50 years old	186 (9)	109 (11)	75 (7)	*P* = 0.007	139 (8)	42 (15)	*P* < 0.001
51–60 years old	92 (5)	54 (5)	38 (4)	n.s.	71 (4)	20 (7)	*P* = 0.016
61–70 years old	39 (2)	22 (2)	17 (2)	n.s.	32 (2)	7 (3)	n.s.
71–80 years old	22 (1)	15 (1)	7 (1)	n.s.	18 (1)	4 (1)	n.s.
81 years or older	^c^	^c^	^c^		^c^	^c^	
Here are some statements about arthritis. For each one, please indicate if you think it is true or false, *n* (%) true							
Arthritis can be cured	179 (9)	115 (11)	63 (6)	*P* < 0.001	109 (6)	68 (25)	*P* < 0.001
There are treatments that help you manage arthritis	1817 (89)	878 (87)	931 (91)	*P* = 0.002	1554 (89)	243 (89)	n.s.
Some types of arthritis can affect your eyesight	588 (29)	258 (26)	327 (32)	*P* = 0.001	505 (29)	80 (29)	n.s.
Blood tests can always confirm a diagnosis of arthritis	533 (26)	267 (26)	265 (26)	n.s.	425 (24)	104 (38)	*P* < 0.001
X-rays can always confirm a diagnosis of arthritis	661 (32)	318 (31)	343 (34)	n.s.	532 (30)	120 (44)	*P* < 0.001

Data weighted to the known population proportions for adults aged 16–75 years in the UK.

a *P*-value comparing male and female respondents.

b *P*-value comparing White with minority ethnic group.

c Data suppressed owing to low numbers (fewer than five per cell) to prevent disclosure.

n.s.: not statistically significant (*P* >0.05).

Respondents were more likely to be aware that the earliest someone could get arthritis is <16 years of age if they had arthritis themselves (60%) or knew someone with arthritis (43%).

However, only 19% of respondents were aware that children <5 years of age can get arthritis. This varied by gender (14% of males compared, with 25% of females) and ethnicity (21% of White, compared with 5% of minority ethnic groups).

### Knowledge about arthritis

Overall, 29% of respondents were aware that some types of arthritis can affect your eyesight; awareness of this extra-articular complication was also higher amongst those with arthritis (34%, *P* < 0.05).

Respondents from ethnic minority groups were also more likely to believe incorrect assumptions about arthritis to be true, such as ‘arthritis can be cured’ (25% compared with 6% of those from white ethnic groups, *P* < 0.05), ‘blood tests can always confirm a diagnosis of arthritis’ (38% compared with 24% of those from white ethnic groups, *P* < 0.05), and ‘X-rays can always confirm a diagnosis of arthritis’ (44% compared with 30% of those from white ethnic groups, *P* < 0.05).

## Discussion

This study has provided the first estimates of the level of community awareness of childhood arthritis in the UK, finding that only 19% of respondents are aware that children <5 years of age can get arthritis. Low levels of community awareness can contribute to delays in attendance at primary care, delays in diagnosis and consequential delays in treatment, hence worse clinical, physical and psychological outcomes. In addition, low levels of community awareness can contribute to stigma and isolation for children and young people with arthritis and their families.

Although the general population are not expected to know the limitations of diagnostic processes to confirm the presence of arthritis, the difference between ethnic groups might be indicative of different levels of overall awareness and might contribute further to delays or misdiagnoses where tests appear normal.

A previous study reported that the incidence and prevalence of JIA is lower amongst minority ethnic groups [9]. This study has found that awareness that children and young people get arthritis is also lower amongst minority ethnic groups and that knowledge of aspects of arthritis is lower amongst those communities. From this study, it is not clear whether low awareness drives reduced presentation to clinical services and lower diagnosis rates or whether lower levels of JIA in ethnic minority groups leads to reduced awareness. Further research into this area is important, with a view to developing targeted awareness-raising resources amongst populations less likely to be aware of childhood arthritis.

The strengths of this study are that it has used a national independent social research organization to produce the first estimates of awareness of childhood arthritis amongst the community. This level of information cannot usually be produced through clinical or academic settings or patient organizations owing to the bias of who usually interacts with those organizations. The biggest limitation is that owing to the sample size, ethnicity can be collected only as a dichotomous variable, and therefore differences between different ethnic groups might be missed; this also limits specific targeting of awareness-raising resources, and further targeted assessment is warranted. All participants were required to be able to read and write English to be able to take part; this might limit the involvement of people for whom English is not their first language.

This study has found that awareness that children and young people can get arthritis is low, potentially contributing to delays in diagnosis of JIA and JIA-related uveitis, delayed treatment and worse clinical and sociological outcomes. Awareness is lower amongst minority ethnic groups; further research to understand this is important to enable targeted interventions and development of awareness-raising resources as appropriate.

## Supplementary Material

rkad099_Supplementary_Data

## Data Availability

The data underlying this article cannot be shared publicly owing to contractual requirements. The data will be shared on reasonable request to the corresponding author.
