# Supplementary material for: Community awareness of childhood arthritis in the UK
Source: Rheumatol Adv Pract. 2024 Jan 22;8(1):rkad099. doi: 10.1093/rap/rkad099 (PMC10849605; doi:10.1093/rap/rkad099)
Supplement: rkad099_Supplementary_Data [file rkad099_supplementary_data.docx]

**Supplementary Data S1. Questions included in the survey**

Q1. As you may know, arthritis is a condition that causes pain and inflammation in a joint.

Which of the following age ranges do you think is the EARLIEST someone can get arthritis?

*We are just interested in your opinion, if you are not sure please give us your best estimate.*

1. Under the age of 5

2. 5 – 10 years old

3. 11 – 15 years old

4. 16 – 30 years old

5. 31 – 40 years old

6. 41 – 50 years old

7. 51 – 60 years old

8. 61 -70 years old

9. 71 – 80 years old

10. 81 years old or above

11. Don’t know

Q2. Here are some statements about arthritis. For each one, please indicate if you think it is true or false.

1. Arthritis can be cured

2. There are treatments that help you manage arthritis

3. Arthritis is common among older people

4. Arthritis can affect children

5. Some types of arthritis can affect your eyesight

6. Blood tests can always confirm a diagnosis of arthritis

7. X-rays can always confirm a diagnosis of arthritis

Q3. Which, if any, of the following statements apply to you?

1. I have arthritis

2. I know a child aged 15 or under who has arthritis

3. I know an adult aged 16 or over who has arthritis

4. I do not know anyone with arthritis

5. Prefer not to say

Demographic variables

Age

Gender

Employment status

Education level

Social grade

Marital status

Household size

Presence of children in household

Household income

Ethnicity
